# An Inducible Transgenic Mouse Model for Immune Mediated Hepatitis Showing Clearance of Antigen Expressing Hepatocytes by CD8+ T Cells

**DOI:** 10.1371/journal.pone.0068720

**Published:** 2013-07-15

**Authors:** Marcin Cebula, Aaron Ochel, Upneet Hillebrand, Marina C. Pils, Reinhold Schirmbeck, Hansjörg Hauser, Dagmar Wirth

**Affiliations:** 1 Model Systems for Infection and Immunity, Helmholtz Centre for Infection Research, Braunschweig, Germany; 2 Mouse Pathology, Helmholtz Centre for Infection Research, Braunschweig, Germany; 3 Gene Regulation and Differentiation, Helmholtz Centre for Infection Research, Braunschweig, Germany; 4 Internal medicine I, University Hospital Ulm, Ulm, Germany; Charite Universitätsmedizin Berlin, Germany

## Abstract

The liver has the ability to prime immune responses against neo antigens provided upon infections. However, T cell immunity in liver is uniquely modulated by the complex tolerogenic property of this organ that has to also cope with foreign agents such as endotoxins or food antigens. In this respect, the nature of intrahepatic T cell responses remains to be fully characterized. To gain deeper insight into the mechanisms that regulate the CD8+ T cell responses in the liver, we established a novel OVA_X_CreER^T2^ mouse model. Upon tamoxifen administration OVA antigen expression is observed in a fraction of hepatocytes, resulting in a mosaic expression pattern. To elucidate the cross-talk of CD8+ T cells with antigen-expressing hepatocytes, we adoptively transferred K^b^/OVA257-264-specific OT-I T cells to OVA_X_CreER^T2^ mice or generated triple transgenic OVA_X CreER^T2^_X_OT-I mice.

OT-I T cells become activated in OVA_X_CreER^T2^ mice and induce an acute and transient hepatitis accompanied by liver damage. In OVA_X_CreER^T2^_X_OT-I mice, OVA induction triggers an OT-I T cell mediated, fulminant hepatitis resulting in 50% mortality. Surviving mice manifest a long lasting hepatitis, and recover after 9 weeks. In these experimental settings, recovery from hepatitis correlates with a complete loss of OVA expression indicating efficient clearance of the antigen-expressing hepatocytes. Moreover, a relapse of hepatitis can be induced upon re-induction of cured OVA_X_CreER^T2^_X_OT-I mice indicating absence of tolerogenic mechanisms.

This pathogen-free, conditional mouse model has the advantage of tamoxifen inducible tissue specific antigen expression that reflects the heterogeneity of viral antigen expression and enables the study of intrahepatic immune responses to both de novo and persistent antigen. It allows following the course of intrahepatic immune responses: initiation, the acute phase and antigen clearance.

## Introduction

The liver is considered a unique solid organ with its ability to cross talk with the immune system and subsequently modulates the intrahepatic immunity. It displays a highly tolerogenic environment to cope with the foreign agents such as endotoxins and food antigens [1,2]. At the same time, it has been well documented that the liver has the remarkable ability to prime immune responses against neo antigens [3] including viral antigens. Spontaneous CD8+ T cell dependent clearance of HBV and HCV infection is achieved in 90% and 20-40% of the cases respectively [4]. However, these infections still contribute major challenge for current medicine. Establishment of chronic infections itself and limitations in treatment efficacy may be a consequence of the tolerogenic properties of the liver [1,2]. Therefore, understanding the mechanisms that control this intricate interplay between intrahepatic T cell responses and the liver environment would be highly advantageous.

To elucidate intrahepatic immunity, transgenic mouse models have been employed that express an antigen constitutively in the liver. However, these models do not allow the induction of a specific endogenous T cell response due to the self-tolerance that arises as a result of thymic expression of the antigen [5,6]. Therefore, these transgenic models have to rely on adoptive transfers of antigen specific T cells to study the liver specific immune responses [5–8]. To circumvent this self-tolerance, mouse models with inducible antigen expression in hepatocytes were established [9,10]. However, reports on immune response in these models are limited.

Alternatively to transgenic models, liver specific expression of antigens has been achieved upon delivery via adenoviral vectors, adeno-associated viral vectors or by hydrodynamic DNA injection. These protocols allow a partial transduction of hepatocytes resulting in de novo mosaic antigen expression [11,12] that reflects the expression pattern observed in natural infections [13]. Both, the viral and the non-viral gene transfer protocols are accompanied by severe inflammation and liver damage [14,15] eliciting additional signals that mask or modulate responses to the antigen presented on hepatocytes. The induction of antigen expression exclusively in hepatocytes without triggering the innate immunity would be a prerequisite to precisely dissect the specific interplay between T cells and hepatocytes.

In most models, a transient immune response to liver specific antigens is obtained, limiting further studies of subsequent chronic inflammatory stages. The establishment of a chronic state with continuous antigen presentation and sustained immunity is a particular challenge.

We aimed at investigating the chronic intrahepatic immunity that is directed against liver restricted antigens. To achieve this, we implemented a conditional mouse model in which ovalbumin (ova) is expressed as a neo antigen in a restricted fraction of hepatocytes resulting in a mosaic expression pattern.

## Results

### Inducible and hepatocyte specific expression of OVA antigen in transgenic mice

**Figure 1:**
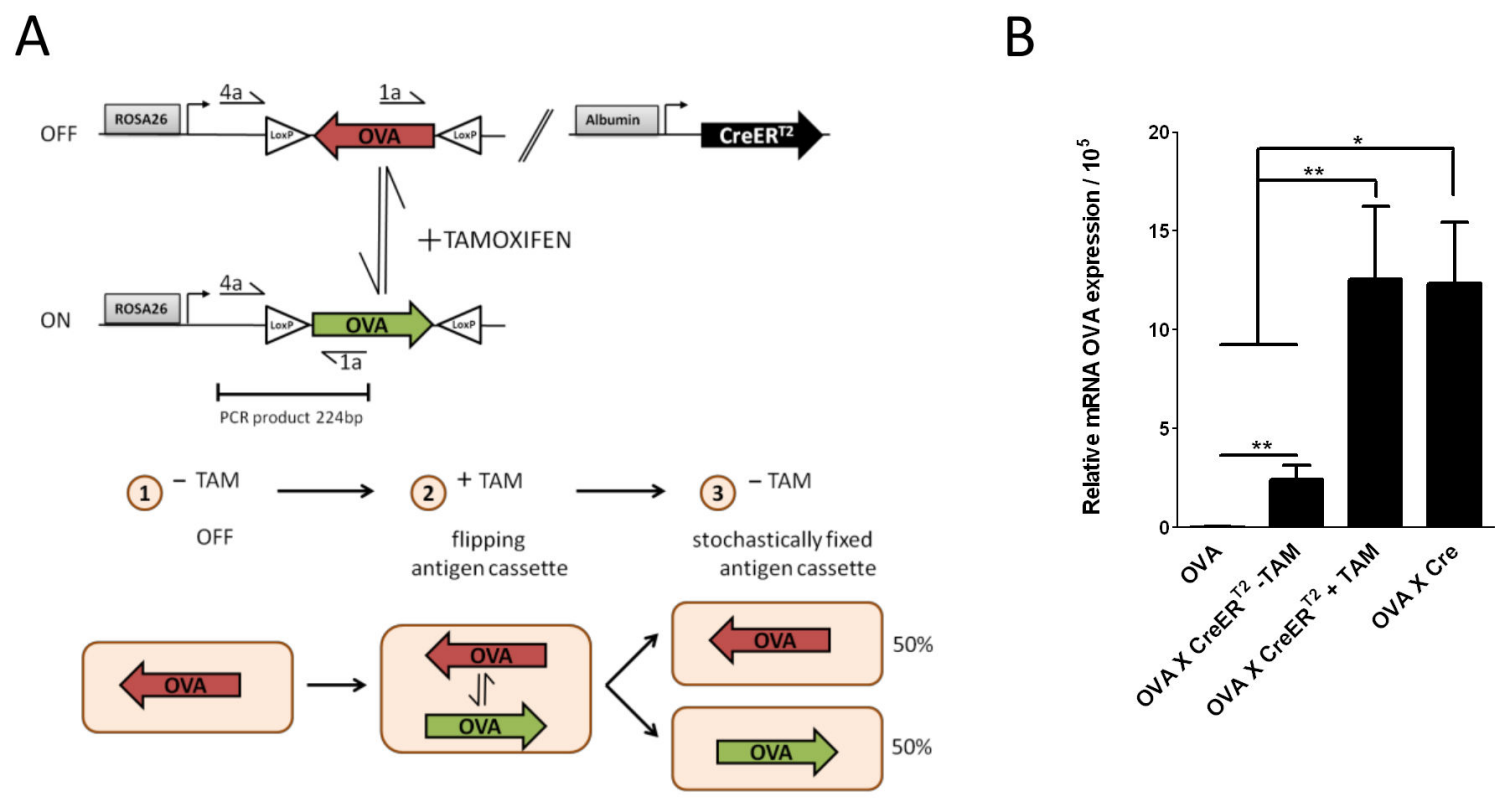
Schematic presentation of antigen induction and its expression levels in the mouse models. **A**. Schematic presentation of the antigen expression system in the mouse models. The OVA cassette is integrated in antisense orientation (OFF-state ‘1’, red) in the ROSA26 locus. The cassette is activated through an Albumin driven expression of CreER^T2^ in hepatocytes by a single application of TAM. Upon binding of TAM, the CreER^T2^ fusion protein translocates to the nucleus and can invert the OVA cassette (ON-state, green). In the presence of TAM, CreER^T2^ continuously flips the OVA cassette and the antigen is stochastically ‘ON’ or ‘OFF’ (‘2’, see also 16). Upon TAM clearance the recombination status becomes fixed (‘3’). Assuming that the activated Cre recombinase is active in all hepatocytes, 50% of cells carry the cassette in the ON-state. The binding sites of primers 1a and 4a used to amplify the OVA cassette in the ON-state are indicated. **B**. OVA mRNA expression levels in the liver of OVA_X_CreER^T2^ mice. OVA_X_CreER^T2^ were treated with a single dose of TAM. 9 days later, the liver was isolated and RNA was subjected to qRT-PCR using the indicated primers. After TAM clearance 50% of all hepatocytes remain in ON-state. The mRNA levels in the non-treated OVA_X_CreER^T2^ mice are 5 fold lower than those from the induced state or the standard. This corresponds to approximately 10% of hepatocytes that express the OVA antigen. OVA_X_Cre mice were used as a standard. Due to the constitutive Cre activity in these mice 50% of all cassettes are supposed to be in the ON-state. The OVA expression levels are related to albumin. The bars reflect samples from 4–8 animals that were individually analyzed.

**Figure 2:**
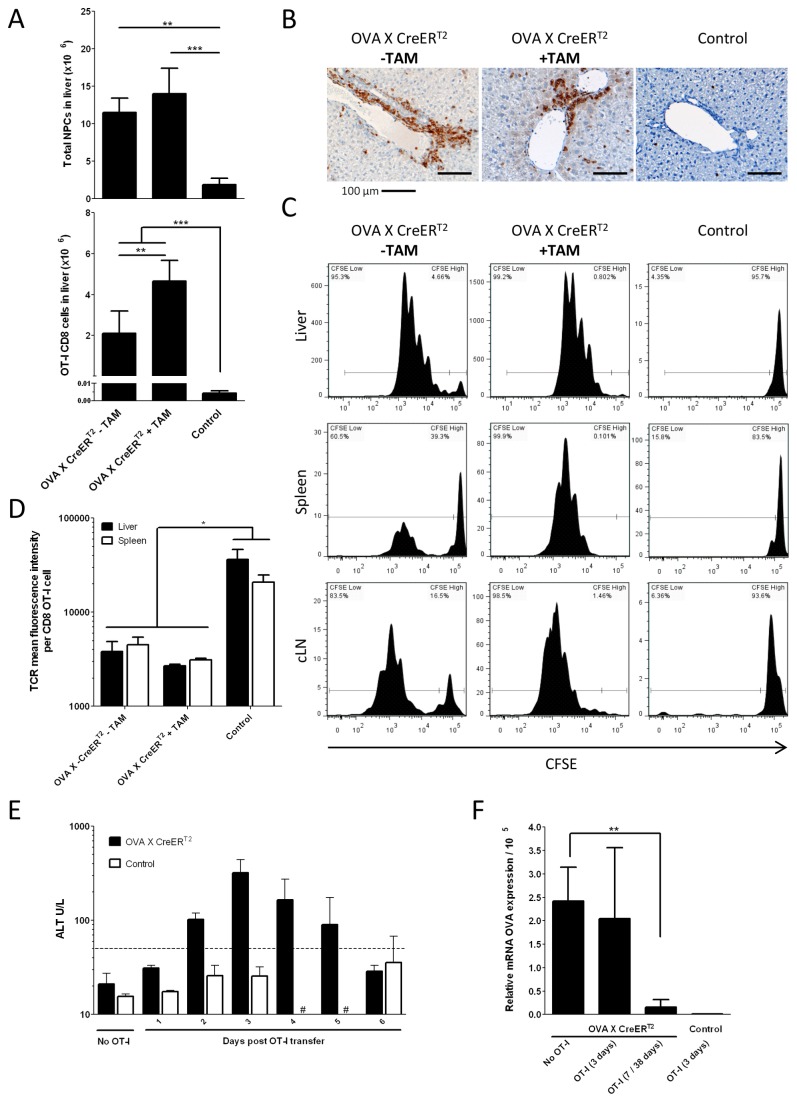
Antigen-driven liver infiltrations and fate of adoptively transferred OT-I T cells. Adoptive transfer of OT-I CD8+ cells to the OVA_X_CreER^T2^ mice either pre-treated with TAM (day -30) or untreated was performed. For analysis mice were sacrificed at day +3 post adoptive transfer. **A**. Accumulation of NPCs and OT-I CD8+ T cells in the liver at day +3 post adoptive transfer (n=6-10). **B**. T cells infiltrating the liver tissue post adoptive transfer. T cells were visualized by indirect immunohistochemical staining using anti-CD3 antibody (brown). Data from representative mice are shown. **C**. Proliferation of OT-I CD8+ T cells quantified by CFSE dilution. Cells were re-isolated on day +3 post transfer. Histograms from representative mice are shown. **D**.T cell receptor down-regulation on OT-I CD8+ T cells as determined by staining with the PE labelled OVA antigen specific tetramers (n=4). **E**. Liver damage assessed by ALT activity measured in blood plasma of OVA_X_CreER^T2^ mice. ALT activity below 40 U/L is considered as physiological, dashed line (n=3-10), # - not done. **F**. Elimination of OVA antigen expressing hepatocytes. ‘ON-state’ OVA mRNA expression in liver samples quantified by qRT-PCR (n=5-8). As control, 4 single transgenic OVA or CreER^T2^ animals were treated with TAM and adoptively transferred with OT-I cells.

**Figure 3:**
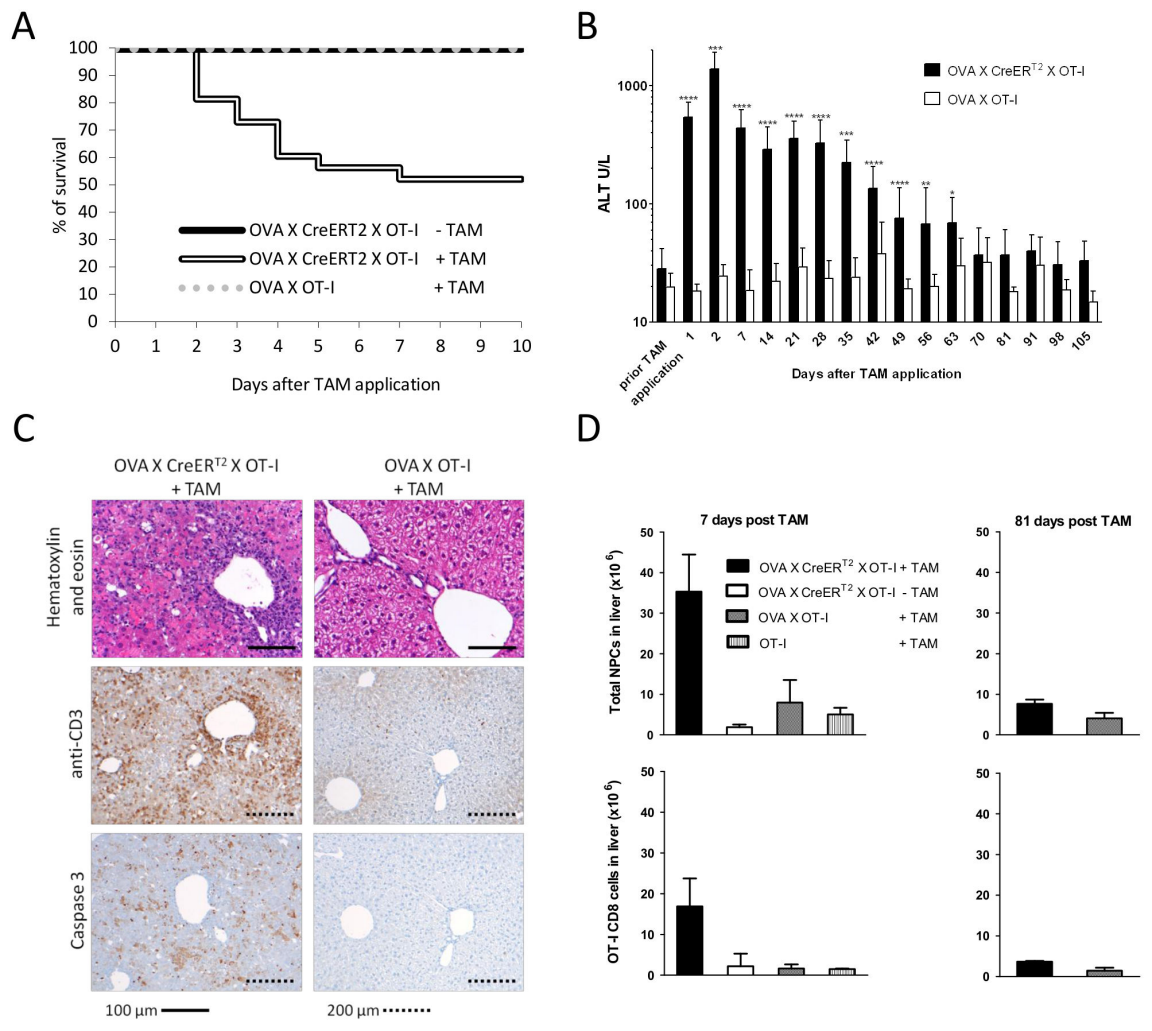
Induction of hepatocyte-specific antigen expression in OVA X CreER^T2^ X OT-I mice. **A**. Survival of OVA_X_CreER^T2^_X_OT-I mice upon treatment with TAM (n=48). **B**. Determination of liver damage assessed by ALT activity. 3-16 animals were analysed per group, statistical significance calculated for groups n≥6. **C**. Histology analysis of liver tissue sections 2 days post TAM application. Sections were stained for T cells (anti-CD3) and apoptotic cells (anti-Caspase 3, brown). **D**. Accumulation of NPCs and OT-I CD8 T cells in the liver at days 7 and 81 post TAM application (n=3-4).

The immunogenic peptide from ovalbumin (ova) was flanked by inversely oriented loxP sites and targeted into the ubiquitously active ROSA26 locus (Figure 1A). In these OVA mice ROSA26-driven ova expression is prevented by integrating the cassette in antisense orientation, creating an ‘OFF-state’ [16]. To achieve hepatocyte specific antigen expression, OVA mice were bred to Alb-CreER^T2^ mice in which the tamoxifen (TAM) inducible Cre recombinase is targeted to the hepatocyte specific albumin gene locus [17]. Feeding the OVA_X_CreER^T2^ mice with TAM activates CreER^T2^ to recombine the ova flanking LoxP sites exclusively in hepatocytes, creating the ‘ON-State’ (Figure 1A and [16]). In the presence of TAM, the flipping of the OVA cassette is continuous (Figures 1A, 2). Once TAM is cleared, an expression status with approximately 50% of hepatocytes in the ‘ON-state’ and 50% of hepatocytes in the ‘OFF-state’ becomes stochastically fixed leading to a mosaic expression pattern of the antigen (Figures 1A, 3). OVA transgenic mice (lacking CreER^T2^ recombinase) show no expression of ova (Figure 1B). TAM treatment of OVA_X_CreER^T2^ mice leads to the induction of ova expression to the same levels as in mice which carry a constitutively active Cre recombinase (Figure 1B). In the non-induced state OVA_X_CreER^T2^ mice display a significantly lower expression level. This basal expression might be due to recombination events of Cre during embryogenesis [18]. Estimating a stochastic expression in 50% of hepatocytes in the ON-state, we calculate that ova is expressed within 10% of hepatocytes in the non-induced OVA_X_CreER^T2^ mice.

### Adoptively transferred OT-I cells infiltrate the liver

To investigate whether the expression of ova mRNA (Figure 1B) is sufficient to induce an immune response, K^b^/OVA257-264-specific OT-I cells (which express the cognate T cell receptor (TCR) directed against the SIINFEKL epitope) were adoptively transferred to OVA_X_CreER^T2^ mice, as well as to OVA control mice. The comparison of the intrahepatic cell frequencies among the recipients confirms infiltration of CD8+ OT-I cells into the liver of OVA_X_CreER^T2^ mice, independent of TAM treatment, whereas the OVA control mice display normal OT-I cell counts in the liver (Figure 2A). Further, histological liver sections of OVA_X_CreER^T2^ mice show the formation of inflammatory foci, contributed by CD3+ cells which are not detectable within OVA control mice (Figure 2B). This confirms that the ova expression in both TAM-treated and non-treated mice sufficient to induce T cell mediated immunity.

### Transfer of OT-I cells causes acute hepatitis

Proliferation and clonal expansion of CD8+ cells is considered to be a prerequisite of potent immunity. Since the liver is known to provide a suppressive milieu which can down-modulate efficient immune responses [1,2] we wondered if adoptively transferred OT-I cells have the potential to mount an effective immune response against ova. Thus, we adoptively transferred CFSE-labelled OT-I cells to non-induced OVA_X_CreER^T2^ mice as well as to TAM induced OVA_X_CreER^T2^ recipients. A decreasing CFSE signal within the OT-I cell population is observed (Figure 2C). As expected, the highest proliferation takes place within the liver of recipients which is the primary site of antigen presence. Proliferation is also observed to a lower extent in the spleen and liver draining lymph nodes of OVA_X_CreER^T2^ mice, whereas transferred cells show no proliferation in any of these tissue of non-expressing control mice (Figure 2C). Another indicator of T cell activation is the down-regulation of the TCR [19]. Indeed, adoptive transfer of OT-I cells to OVA_X_CreER^T2^ mice, but not to OVA mice significantly reduces the TCR expression of OT-I cells confirming specific activation in an antigen dependent manner (Figure 2D).

The proliferation of OT-I cells upon transfer to OVA_X_CreER^T2^ mice is similar in the liver and independent of TAM treatment (Figure 2C). Adoptive transfer of 5*10^6^ OT-I cells results in an acute hepatitis, starting 2 days after adoptive transfer as indicated by increased serum alanine aminotransferase (ALT) activity. In Figure 2E, the liver damage is shown for non-induced mice for a period of 6 days. ALT values peak at day +3 post adoptive transfer and decrease to physiological levels at day +6 (Figure 2E). The qRT-PCR determination of ova expression during this period confirms the presence of the antigen at day +3 upon transfer to the same level as before transfer of OT-I cells (Figure 2F). Importantly, ova mRNA levels are found to be reduced to the level of OVA control mice at later time points (Figure 2F). This indicates that the adoptively transferred T cells are efficiently activated, resulting in clearance of antigen presenting hepatocytes.

### Induction of fulminant and chronic hepatitis

To study the immune response in presence of endogenous OT-I cells we created OVA_X_CreER^T2^_X_OT-I mice. These mice display normal counts and activity of OT-I cells and no signs for establishment of tolerogenic mechanisms (data not shown). Upon TAM administration and activation of hepatocyte specific ova antigen expression, 50% of the mice die within a few days (Figure 3A). The surviving animals show a fulminant hepatitis accompanied by highly elevated levels of ALT and bilirubin already on the first day post induction (Figure 3B and data not shown). At day 2, during the initial phase of the intrahepatic immune response, numerous severe necro-inflammatory infiltrations are observed which are largely associated with CD3+ T cells (Figure 3C). Further, histological analysis revealed Caspase-3 positive parenchymal cells indicating apoptotic hepatocytes. The severe hepatitis is also reflected by an increase of the non-parenchymal cell population and a high number of OT-I cells in liver in the acute phase (Figure 3D).

After this acute phase the mice develop a sustained hepatitis accompanied by inflammatory cell infiltrations (Figure S1A) and elevated levels of ALT lasting for 9 weeks post induction (Figure 3B). Upon recovery (at day 81) the T cell infiltrations and also the total nonparenchymal cell (NPC) population are significantly reduced and comparable to control mice (non-induced mice or mice lacking Cre) (Figure 3D and Figure S1B) indicating a complete recovery from hepatitis in these animals.

### Clearance and reactivation of antigen expressing hepatocytes

**Figure 4:**
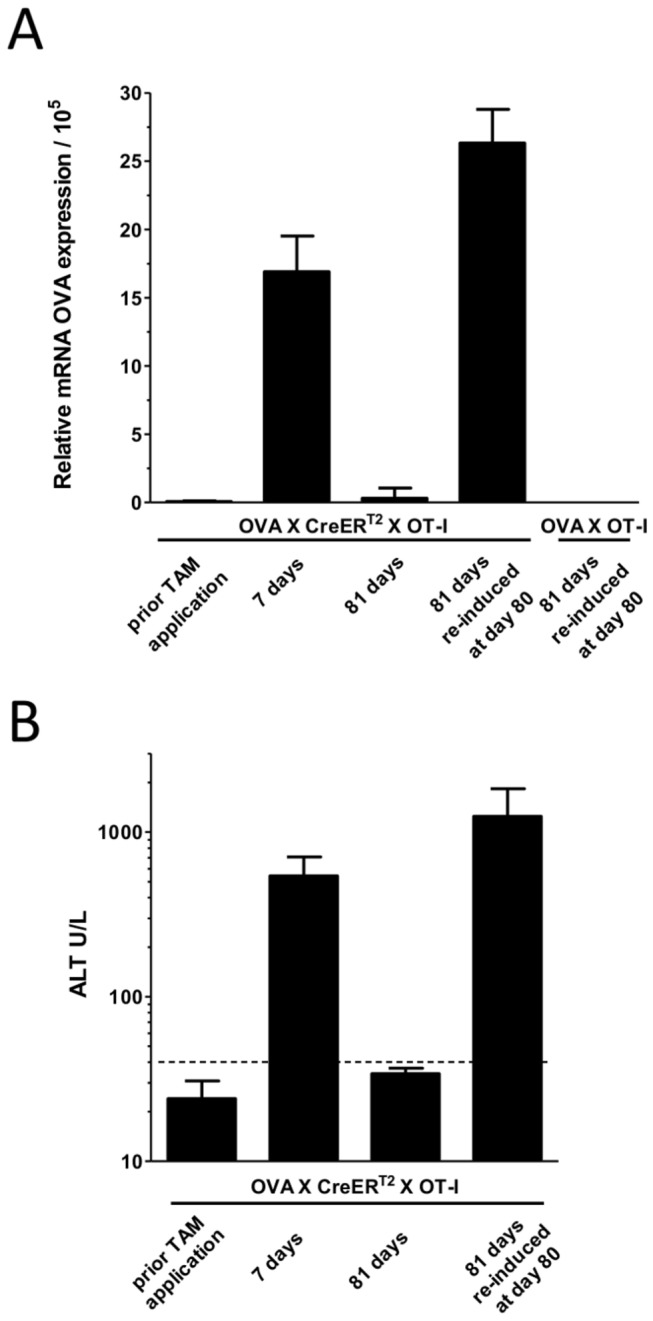
Elimination and re-induction of OVA expressing hepatocytes in OVA_X_OT-1 mice. OVA_X_CreER^T2^_X_OT-I mice were induced with TAM and monitored for OVA antigen expression (qRT-PCR, **A.**) and liver damage (ALT levels, **B.**). One experimental group was re-induced with TAM at day 80 (n=3).

We investigated the ongoing immune response against hepatic antigens and observed how liver injury is finally resolved. Different mechanisms could be expected to be involved. These include the induction of mechanisms that impair T cell action (exhaustion), T cell deletion or the formation of regulatory T cells. As well antigen clearance could have been achieved, as observed upon adoptive transfer of cells to the non-induced OVA_X_CreER^T2^ mice (Figure 2F). Thus, antigen expression levels were monitored. Intriguingly, OVA_X_CreER^T2^_X_OT-I mice show no basal ova mRNA expression before TAM feeding (Figure 4A). At day seven upon induction, ova mRNA levels were comparable to induced OVA_X_CreER^T2^ or OVA_X_Cre double transgenic animals (compare to Figure 1B). Interestingly, when hepatitis is resolved at day 81 post TAM feeding in OVA_X_CreER^T2^_X_OT-I the level of ova mRNA is found to be as low as prior to TAM feeding or in the control animals. The experiments prove a complete clearance of antigen (Figure 4A, B) and suggest that antigen elimination is crucial for recovery. Thus, the observed immune response in induced OVA_X_CreER^T2^_X_OT-I mice is sufficiently potent to eliminate antigen expressing hepatocytes.

**Figure 5:**
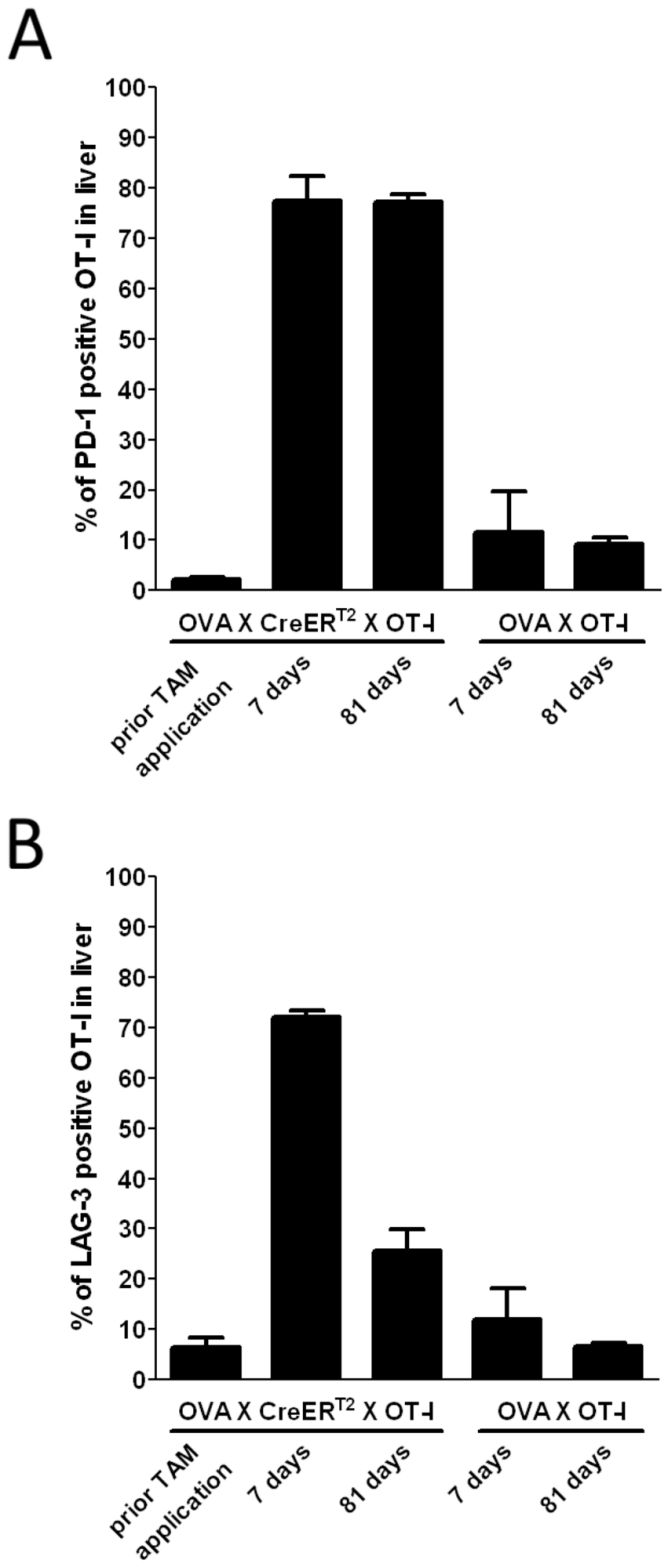
Expression of exhaustion markers in induced OVA X CreERT2 X OT-I mice. **A**. PD-1 and **B**. LAG-3 expression on hepatic OT-I CD8 T cells assessed by FACS analysis prior and post TAM (n=3-4).

In chronically infected patients T cells have been found to display sustained expression of exhaustion markers [20,21]. This is reflected in the OVA_X_CreER^T2^_X_OT-I model by high frequencies of OT-I cells expressing the co-inhibitory receptors PD-1 and LAG-3 (Figure 5A, B and Figure S2A, B), even after recovery from hepatitis and antigen clearance.

The OVA_X_CreER^T2^_X_OT-I model provides the option of re-induction of antigen expression thus mimicking re-occurrence of viral gene expression. Indeed, upon a second application of TAM at day 80, i.e. when the mice have recovered from initially induced hepatitis and have cleared ova expressing cells, re-expression of antigen is observed (Figure 4A). Importantly, the re-induction of antigen expression is accompanied by a relapse of hepatitis at day 81 (Figure 4B), indicating a potent activation of T cells also at this time point.

## Discussion

A crucial role of potent intrahepatic CD8^+^ T cells in infection scenarios has been well documented [22–24]. However, the effector phenotype of CD8+ T cells primed in the liver and the resulting response remains largely controversial [25,26].

In this study, we show and quantify the consequences of the intrahepatic T cell activation and the effector function of cytotoxic T cells within the suppressive milieu of the liver. We used two experimental settings, adoptive transfer of OT-I cells to OVA_X_CreER^T2^ mice and triple transgenic OVA_X CreER^T2^_X_OT-I with an endogenous supply of OT-I cells. The results indicate that in both experimental conditions, OT-I cells acquire a cytotoxic phenotype and are capable to eliminate target hepatocytes.

The overall expression level of ova in this model is low and can only be determined on mRNA level by qRT-PCR. If compared to actin, ova is expressed about 80-100fold lower in the liver tissue (data not shown). Still, this expression level is sufficient to activate the specific T cells. Upon adoptive transfer of OT-I cells to non-induced and induced OVA_X_CreER^T2^ mice, the transferred T cells proliferate and accumulate in the liver (Figure 2A, C), leading to transient liver damage as reflected by ALT increase. The induced OVA_X_CreER^T2^ mice display also hepatitis with similar kinetics and slightly lower ALT values in comparison to the non-induced mice (data not shown). By induction of OVA antigen in OVA_X_CreER^T2^_X_OT-I a strong T cell response is provoked, leading to immune mediated fulminant hepatitis, which is lethal in every second animal. Surviving mice display a sustained hepatitis which lasts for about 9 weeks. The recovery correlates with an efficient clearance of antigen expressing hepatocytes. This is remarkable since pathogen clearance upon hepatotropic viral infections is frequently found to be impaired by the establishment of tolerogenic mechanisms [27,28]. An up-regulation of exhaustion markers is detected in T cells which is in accordance with comparable observations in patients. However, formation of regulatory T cells could not be observed (data not shown).

One important feature of the OVA_X_CreER^T2^ model is the fact that antigen expression is induced only in a fraction of hepatocytes as a consequence of its stochastic induction mode (Figure 1A). Upon single feeding with TAM, 50% of the hepatocytes are expected to display the antigen. Due to the low antigen expression level this could not be confirmed by immune histology. However, this mosaic type of antigen expression is functionally proven in OVA_X_CreER^T2^_X_OT-I mice by the complete elimination of antigen expressing hepatocytes once hepatitis is resolved (Figure 4A, B). The remaining hepatocytes in the ‘OFF-state’ could proliferate, repopulate the liver and rescue the animal. These ‘OFF-state’ hepatocytes retain the ability to re-activate expression upon TAM treatment which is confirmed by the efficient re-induction of the OVA antigen in the repopulated liver. The mosaic expression type of ova mimics the randomly infected hepatocytes in humans which can be targeted and eliminated once a functional immune response is established. The model presented here is unique in providing this expression phenotype under sterile conditions, excluding secondary signals as elicited by viral vector infection or high volume tail vein injection of DNA. In this regard, this reflects the natural situation after infection with HBV in which no induction of innate immune response genes is observed [29].

The model presented here offers the possibility to evaluate treatment protocols designed to suppress excessive immune reactions or to avoid severe liver injury. Upon antigen re-induction in OVA_X_CreER^T2^_X_OT-I mice, an immune response to a re-emerging antigen is mimicked which allows to study aspects of re-activation of virus antigen expression or the reinfection event. Moreover, the model could be of value to study novel strategies aiming at boosting and restoring of the intrahepatic immunity against hepatocytes specific pathogens. This includes immune-modulatory treatments or application of therapeutic vaccines.

Together, the OVA_X_CreER^T2^_X_OT-I model provides a novel tool to investigate T cell responses in highly controlled, synthetic and pathogen-free conditions. It reflects the course of immune response including the acute and long lasting phase of hepatitis and its final resolution.

## Materials and Methods

### Ethics Statement

All animal experiments have been performed in accordance with German Animal Welfare Law, institutional guidelines and have been approved by the local government of Lower Saxony (animal permission 33.14-42502-04-10/199). To minimize the suffering of the animals, mice displaying severe hepatitis with decreased vitality were euthanized by isoflurane treatment.

### Animals, ova antigen induction and ALT measurement

All mice used in the experiments were of the C57Bl6/J background. OVA transgenic mice expressing ova peptide (aa 246–353) containing MHC class I and II epitopes fused to GFP, have been previously described and characterized as ROSAOVA mice in [16]. OVA_X_CreER^T2^ mice were obtained by mating OVA mice with Alb-CreER^T2^ mice [17]. Constitutive OVA expressing mice (OVA_X_Cre) were generated by crossing OVA mice to Alb-Cre mice [30]. OVA_X_CreER^T2^_X_OT-I mice were generated by breeding OVA_X_CreER^T2^ mice with OT-I mice [31]. Animals were bred and kept in individually ventilated cages under specific pathogen free conditions. All mice were bred and kept in in-house animal facilities and were used at the age of 8 to 16 weeks.

Antigen expression was induced by application of 1 mg Tamoxifen (TAM), (Ratiopharm, Ulm, Germany) in 400 µl ClinOleic (Baxter, Lessines, Belgium) by oral gavage. In an alternative protocol, two doses of 8mg TAM were administered resulting in the same OVA expression levels and kinetics of expression. Blood samples were taken by retro-orbital bleeding and mixed 1:4 with Heparin 1,25I.E. (Ratiopharm). Determination of ALT was carried out from 32 µl of plasma using a Reflovet®Plus reader (Roche Diagnostics, Mannheim, Germany).

### Total cell isolation and staining

Mice were sacrificed by treatment with IsoFlo® (Abbott animal health care, Illinois, USA). Spleens were isolated, minced through a sieve, and erythrocytes were lysed. Lymphocytes were separated by a 100 µm cell strainer (Becton). For adoptive transfer experiments, CD8+ cells were purified by negative selection using a CD8a+ T cell isolation kit II (Miltenyi, Germany) according to manufacturer’s instructions. To monitor T cell proliferation CD8+ T cells were labelled with carboxyfluorescein succinimidyl ester (CFSE) (CellTrace CFSE Cell Proliferation Kit, Invitrogen life technologies according to manufacturer’s instructions.

Cells from liver draining lymph nodes were isolated by mincing lymph nodes through a 100 µm cell strainer (Becton).

Livers were perfused in situ with 10 ml Liver Perfusion Medium (Invitrogen life technologies 17701-038, UK) and samples were isolated for RNA isolation and histology. To isolate lymphocytes, 5 ml Liver Digest Medium (Invitrogen life technologies 17701-034, UK) was injected via the portal vein. Pre-digested livers were smashed and incubated in Liver Digest Medium for 30 min. at 37°C before mincing through a 100 µm cell strainer. Cell suspensions were separated by centrifugation (5 min at 500 rpm). While the parenchymal fraction containing hepatocytes sedimented, the NPC fraction was collected from the supernatant and laid on a 70% Percoll Separating Solution (Biochrom, Berlin, Germany) and centrifuged 20 min at 2000 rpm without brake. Cells were collected, erythrocytes were lysed and the leukocytes were washed [32]. All washing steps were done in PBS/1% FCS.

### Immuno-histochemical analysis

Liver samples were fixed in 4% formalin (pH 7,4), dehydrated and embedded in paraffin. 3 µm thick sections were stained with Hematoxylin-Eosin, anti-CD3 antibody (Neo Makers, RM-9107-S1, Clone SP7), or anti Caspase3 (Cell signalling, clone Asp175). Goat anti rabbit antibody conjugated with Horseradish peroxidase (Thermo Scientific, TR-060-BN) was used as secondary antibody, 3,3'-Diaminobenzidine (DAB) was used as chromogen. Blinded samples of sections were evaluated.

### RNA isolation and qRT-PCR

Harvested liver samples were stored in All Protect Tissue Reagent (Qiagen, Hilden, Germany). Samples were homogenized in RLT-Buffer (MP FastPrep®-24 device) and purified using the RNeasy-kit (Qiagen, Hilden, Germany) according to the manufacturer’s instructions, including a DNase-digestion step. 2 µg of total RNA was reverse transcribed using Ready-To-Go^TM^ You-Prime First-Strand Beads (GE Healthcare, Buckinghamshire, UK). For quantitative RT-PCR, Ova-gene cDNAs were amplified using primer pair 1a (5’-CAGGCACTCCTTTCAAGACC) and 4a (5’-GCGGTTGAGGACAAACTCTT) which is specific for the ‘ON-state’ (Figure 1A). The number of Ova-specific gene transcripts was related to albumin transcripts. qPCR was performed in triplicates from individual mice. Normalized values are depicted.

### Flow cytometry

Isolated immune cells were stained with the fluorescently labelled monoclonal anti-mouse antibodies anti-CD8PerCP-Cy5.5, anti-PD-1-FITC, anti-LAG-3-PE and anti-Thy1.1-PE (e-Bioscience, San Diego, CA). The OVA specific TCR cells were stained with iTAg-PE H2Kb Ova-SIINFEKL Tetramer (Beckman Coulter). Antibodies were diluted in PBS/2% FCS. Prior to staining, the Fc receptor was blocked. Analysis was performed using FACS LSRII (Becton Dickinson) and FlowJo-software (TriStar Inc, USA).

### Statistical analysis

Data are represented as mean of 3-16 biological replicates and standard deviations are indicated. Mann Whitney test was used for comparison of two data sets of n≥4. Differences between sets of data were considered to be statistically significant for P-values *p<0,05, **p<0,01, ***p<0,001, ****p<0,0001.

## Supporting Information

Figure S1 Hematoxylin and eosin stained liver sections of OVA X CreERT2 X OT-I and control OVA_X_OT mice. Mice were sacrificed on day 42 post Tamoxifen application during the phase of ongoing/chronic hepatitis (**A**) and on day 81 post Tamoxifen application after the recovery form hepatitis (**B**).
(TIF)

Figure S2 Expression of exhaustion markers in OVA X CreERT2 X OT-I mice and control OVA_X_OT mice. T cells were isolated prior to Tamoxifen application and day 7 and 81 afterwards. FACS histograms of PD-1 (**A**) and LAG-3 (B) expression on hepatic OT-I CD8 T of representative mice are shown.
(TIF)
