# Supplementary figures and images for: An Inducible Transgenic Mouse Model for Immune Mediated Hepatitis Showing Clearance of Antigen Expressing Hepatocytes by CD8+ T Cells
Source: PLoS One. 2013 Jul 15;8(7):e68720. doi: 10.1371/journal.pone.0068720 (PMC3711822; doi:10.1371/journal.pone.0068720)

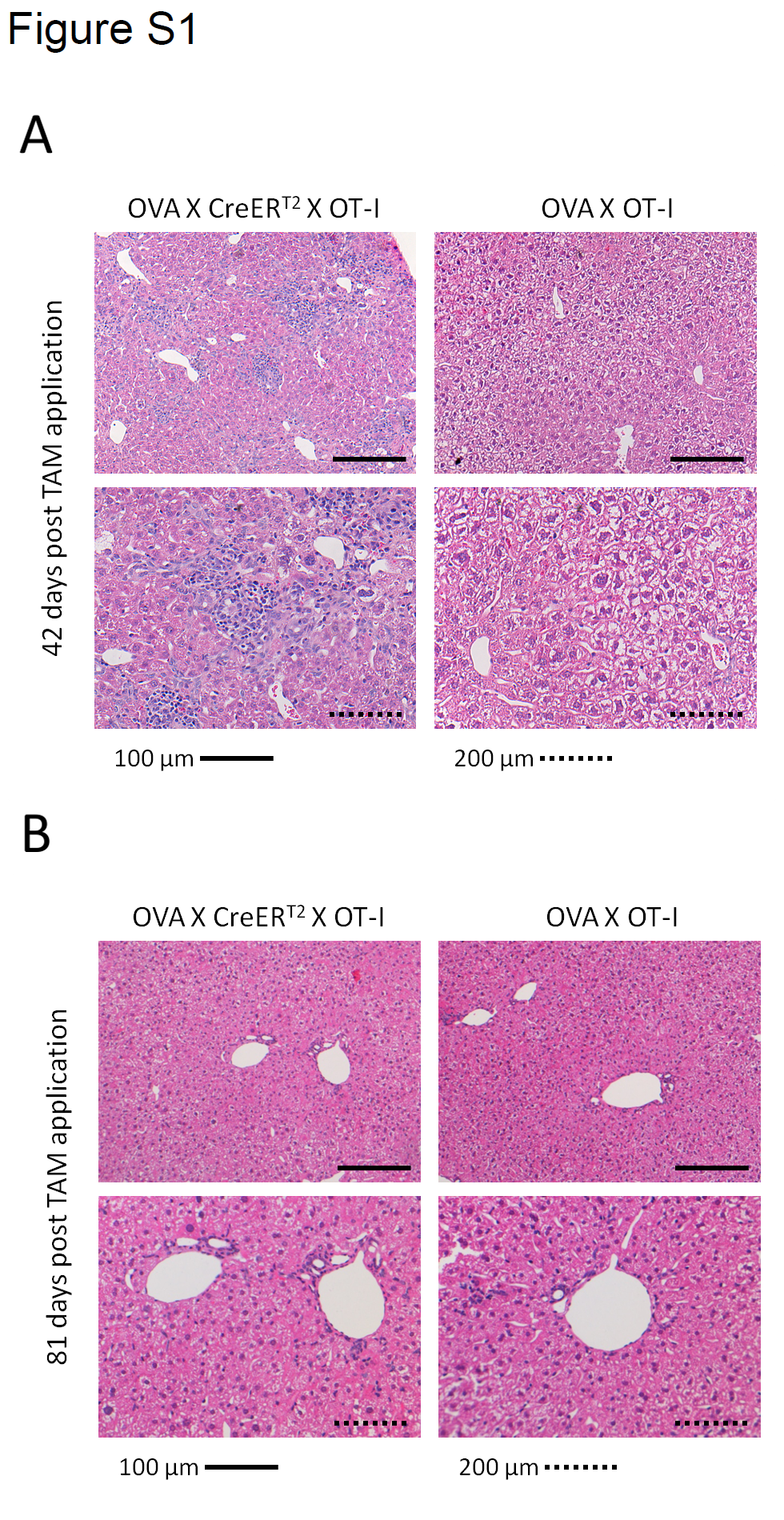

Supplement: Figure S1 — Mice were sacrificed on day 42 post Tamoxifen application during the phase of ongoing/chronic hepatitis (A) and on day 81 post Tamoxifen application after the recovery form hepatitis (B). (TIF) [file pone.0068720.s001.tif]

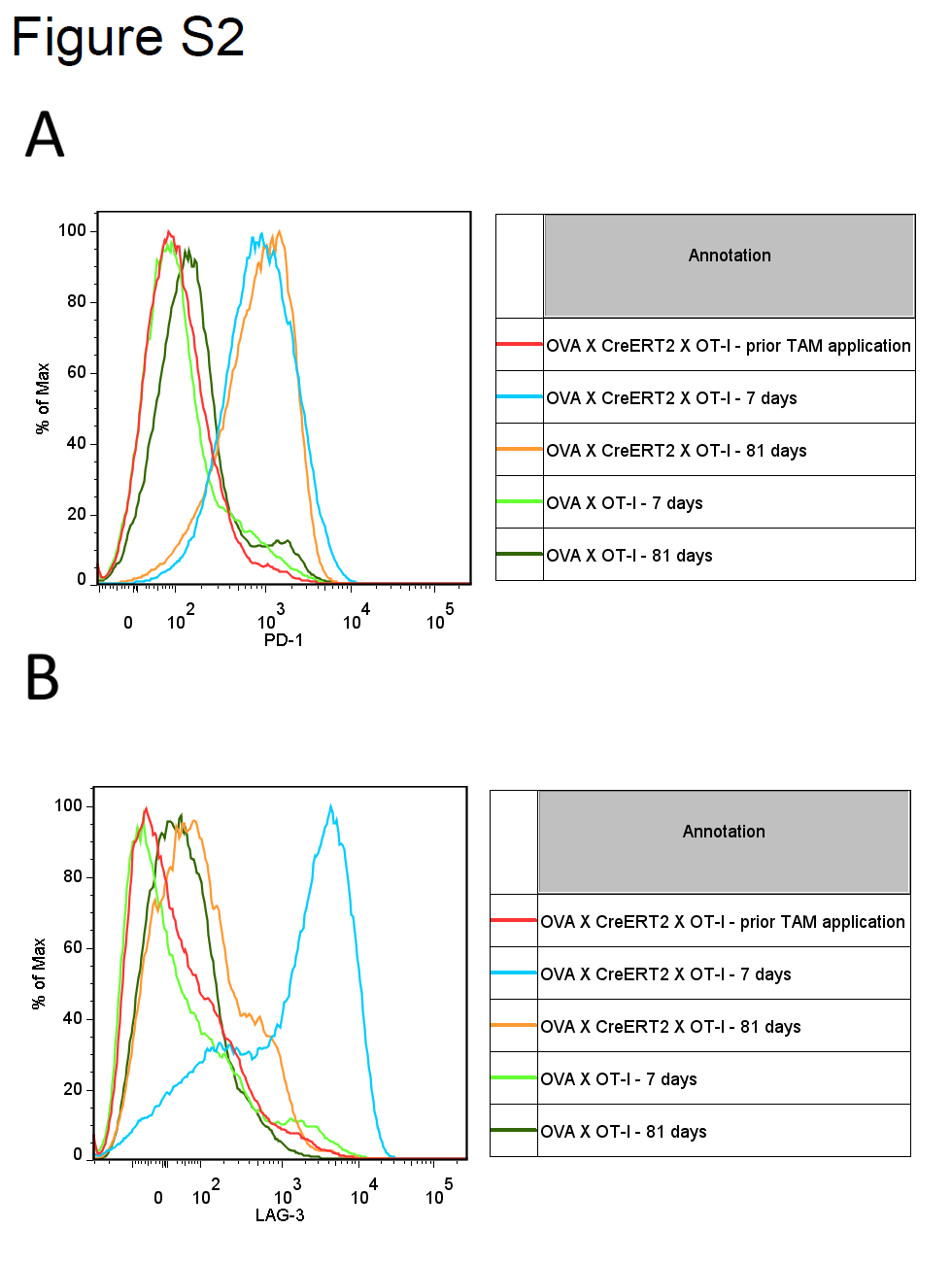

Supplement: Figure S2 — T cells were isolated prior to Tamoxifen application and day 7 and 81 afterwards. FACS histograms of PD-1 (A) and LAG-3 (B) expression on hepatic OT-I CD8 T of representative mice are shown. (TIF) [file pone.0068720.s002.tif]
